# Differentially Expressed miRNAs in Acute Wound Healing of the Skin: A Pilot Study

**DOI:** 10.1097/MD.0000000000000458

**Published:** 2015-02-20

**Authors:** Ping Li, Quanyong He, Chengqun Luo, Liyuan Qian

**Affiliations:** From the Department of Burns and Plastic Surgery (PL, QH, CL); and Department of Thyroid and Breast Surgery (LQ), The Third Xiangya Hospital, Central South University, Changsha, Hunan, PR China.

## Abstract

The aim of the present study was to compare expression of microRNAs (miRNAs) from scar and normal skin areas in patients who suffered acute injuries in the skin.

A total of 9 patients with acute injuries in the skin who received surgical treatment from December 2012 to March 2013 were included in this pilot study. Specimens from the hypertrophic scar and normal skin areas were obtained from the same patient during surgery. To screen for differentially expressed miRNAs, we applied 3 statistical methods, namely the traditional *t* test, the false discovery rate (FDR), and a novel sure independence screening procedure based on the distance correlation (DC-SIS). We examined the functional trends and metabolic and regulatory pathways for the target genes of the identified miRNAs, and explored interaction of these miRNAs in the implication of scar healing using Ingenuity Pathway Analysis.

DC-SIS identified 18 differentially expressed miRNAs, 4 of which (miR-149, miR-203a, miR-222, miR-122) were also identified by FDR. The target genes of the 4 miRNAs exhibit a variety of biological functions, and are involved in various pathways such as mitogen-activated protein kinase, Wnt signaling, and focal adhesion. We identified 1 network in which 14 out of the 18 differentially expressed miRNAs were involved. Many of the miRNAs in the network target genes were involved in cell proliferation and apoptosis.

In this pilot study, we identified several miRNAs exhibiting differential expression in patients who suffered acute injuries in the skin. Further studies on these miRNAs are needed to validate our findings and explore their roles in the wound healing process of the skin.

## INTRODUCTION

In adult humans the skin is the largest organ and has various functions including barrier defense, UV protection, thermoregulation, pigmentation, sensation of touch and pain, and regulation of water loss from the epidermis.^1^ Acute wounds in the skin caused by accidents such as burning or trauma are serious injuries. Wound healing in the skin is a dynamic process in which various types of cells, such as cells involved in acute and chronic inflammation,^2^ are involved.

MicroRNAs (miRNAs) refer to a class of single-stranded RNAs that are 19 to 24 nucleotides in length. They suppress the expression of target genes by messenger RNA (mRNA) degradation or the blockade of mRNA translation by binding to the 3′-untranslated region of target mRNA.^3^ One individual miRNA could regulate many genes, and similarly 1 individual gene could also be regulated by more than 1 miRNA.^4,5^ miRNAs are reported as critical regulators in skin morphogenesis, wound healing, and regeneration by controlling proliferation, differentiation, and apoptosis of skin cells.^6^ However, little is known about the key miRNAs that are involved in acute wound healing in the skin and their biological targets and functions, partially due to the dynamic interaction between multiple cell types during wound healing.

To identify the critical miRNAs in patients with acute skin injuries, we compared the miRNA expression from scar and normal skin cells of the same patients. We conducted network analysis of the identified miRNAs showing differential expression, and explored their potential target genes and performed Gene Ontology (GO) and Kyoto Encyclopedia of Genes and Genomes (KEGG) analysis of these target genes.

## MATERIALS AND METHODS

### Participants

A total of 9 patients were included for this study. All of them received surgical treatment during the period from December 2012 to March 2013 in the Department of Burns and Plastic Surgery of The Third Xiangya Hospital of Central South University in China. Age of these patients ranged from 3 to 43. All of them received no medical or radiological therapy before surgery, and had no history of diabetes, hypertension, liver, or other chronic diseases. Informed consent was obtained from all patients or their closest relatives. This study was approved by the ethical committee of the Central South University.

### Resection of Tissue Specimens

Specimens from the hypertrophic scar and normal skin areas were obtained from the same patient during surgery. All the specimens were obtained at least 6 months after healing of wound surface. In the surgery, the scar was removed and cut into 2 × 2 cm. Specimens from the normal skin were obtained in areas at least 1 cm away from the scar areas. Both types of specimens were immediately put in liquid nitrogen and kept at −80°C.

### miRNA Microarray and Hybridization

miRNAs were extracted using the miRcute RNA Isolation Kit (Tiangen Biotech, Beijing, China). Quality control, labeling, and hybridization were performed commercially according to protocols in the μParaflo microRNA microarray assay (LC Sciences, Hangzhou, Zhejiang, China). Fluorescence images were collected using a laser scanner GenePix 4000B (Molecular Device, Sunnyvale, CA) and digitized using Array-Pro image analysis software (Media Cybernetics, Rockville, MD). Data were transformed by first subtracting the background and then normalizing the signals using a locally weighted regression filter.^7^

### Statistical Analysis

We used 3 methods to screen for differentially expressed miRNAs. First, we applied the traditional *t* test and set statistical significance level at a *P* value ≤0.05 (criterion 1). We then used false discovery rate (FDR) to adjust for multiple testing and considered a change to be significant if the corresponding q-value is ≤0.05 (criterion 2). Finally we applied a newly developed statistical screening method, a sure independence screening procedure based on the distance correlation (DC-SIS; criterion 3).^8^ As a novel statistical method for feature selection for ultrahigh-dimensional data, DC-SIS is model free since it makes no assumption (eg, linear model) for the response (eg, miRNA expression) and the predictors (eg, presence of scar or not). DC-SIS therefore is robust to model misspecification, and the sure independence feature it bears ensures that all truly important variables can be selected with sufficient sample size. DC-SIS therefore is more flexible and reliable in selecting important predictors than conventional statistical methods such as the *t* test. Because our sample size is limited, to reduce the possibility of missing important miRNAs, we chose our model size to be 6[n/log(n)], where n is the sample size and [n/log(n)] denotes the integer part of n/log(n).

Statistical analyses were performed using the program R (www.R-project.org) and SAS version 9.3 (SAS Institute Inc, Cary, NC).

### Function Annotation and Pathway Analyses

We examined the functional trends and metabolic and regulatory pathways for the target genes of the top-hit miRNAs identified in our study (see below for details). Biological targets of the miRNAs were predicted using the online web server TargetScan (http://www.targetscan.org/). Functional GO analysis and KEGG analysis were performed using the online gene set analysis toolkit WebGestalt (http://bioinfo.vanderbilt.edu/webgestalt). GO analysis includes 3 parts, namely the biological process, molecular function, and cellular component. Gene overrepresentation was identified using hypergeometric distribution. Multiple testing was adjusted for using the FDR. For simplicity, we focus on the top 10 most significant GO categories and KEGG pathways.

### Ingenuity Pathway Analysis

To further explore the relationship of the 18 identified miRNAs, we used Ingenuity Pathway Analysis (IPA, QIAGEN, Redwood City, CA, http://www.qiagen.com/ingenuity) to construct molecular networks to better understand the interaction of these miRNAs in the implication of scar healing. IPA constructs networks based on Ingenuity Knowledge Base (IKB), a repository of millions of expert-curated findings of biological and chemical interactions and functional annotations. Each individual interaction in the network was supported by findings in Ingenuity Knowledge Base. Each identified network has a p-score (−log_10_[*P* value]), where the *P* value was calculated using Fisher exact test, representing the probability of finding no less than the number of focus miRNAs (out of all the 18 miRNAs).

## RESULTS

### Differentially Expressed miRNAs

A total of 513 miRNAs were interrogated and included in the final analyses, and we found that 170 (33.1%) had significant changes with corresponding *P* values ≤0.05 using the *t* test. Specifically, 58 miRNAs (34.1%) exhibited elevated expression, and 112 (65.9%) exhibited lowered expression (Table 1).

**Table 1:**
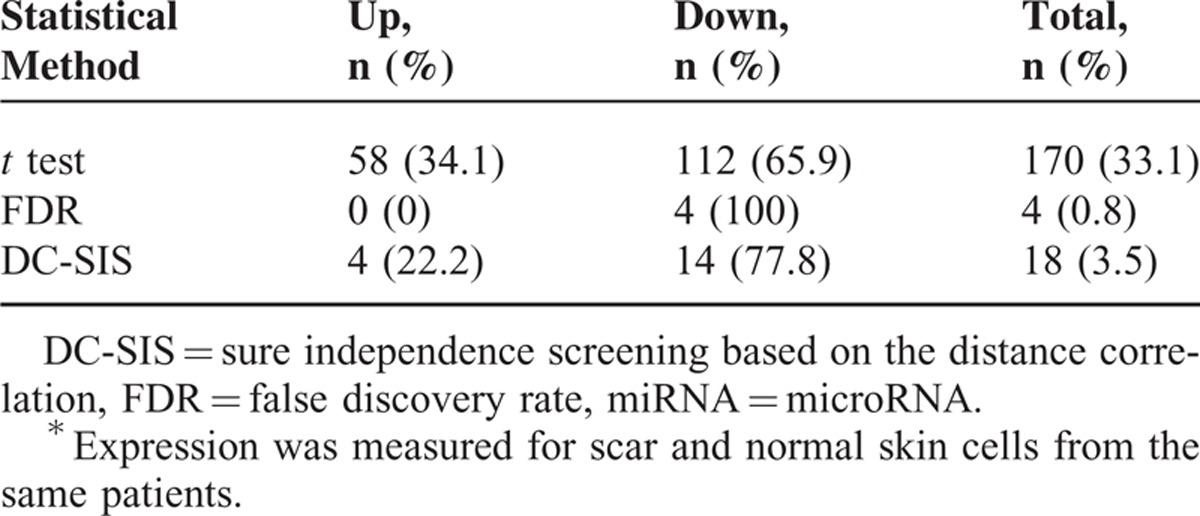
Summary of Differentially Expressed miRNAs^∗^

After adjusting for multiple testing using FDR, we found that only 4 miRNAs (miR-149-5p, miR-203a, miR-222-3p, miR-122-5p) had significant change in expression. Specifically, none exhibited elevated expression, and all the 4 miRNAs exhibited lowered expression (Table 1).

DC-SIS feature screening identified 18 miRNAs, all of which had small *P* values of change in expression using paired *t* test (Table 2). Specifically, 4 miRNAs (22.2%) exhibited elevated expression, and 14 miRNAs (77.8%) exhibited lowered expression (Table 1). All the 4 miRNAs identified by FDR were also identified by DC-SIS, and hence are those showing significant changes in expression as identified by all the 3 criteria (Table 2).

**Table 2:**
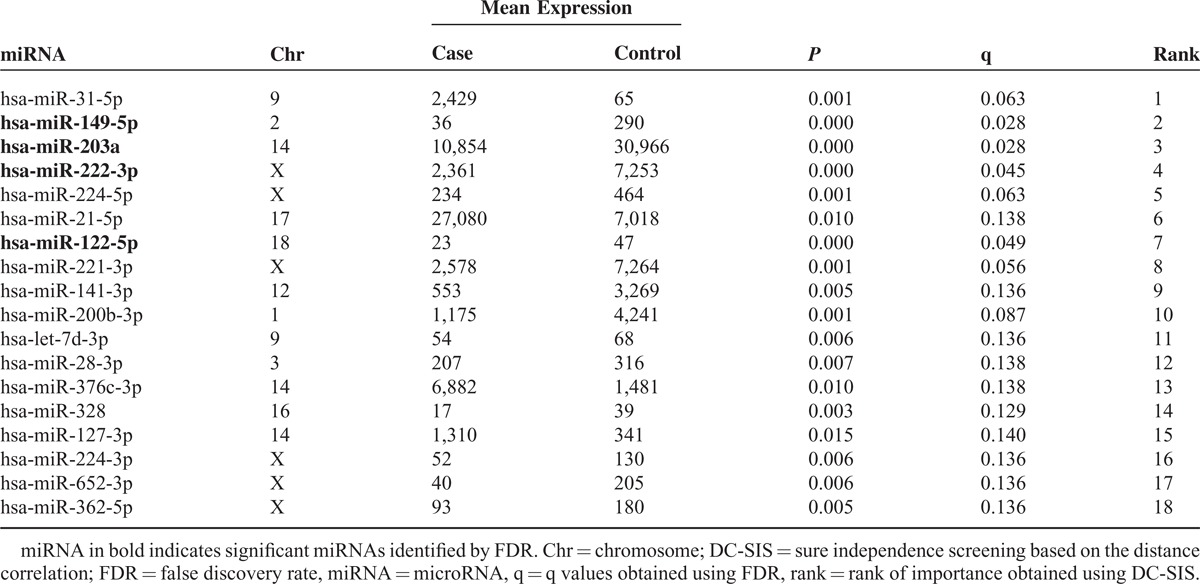
Information of the 18 miRNAs Identified by DC-SIS

### Functional and Network Analysis

We explored functions of the target genes for the 4 miRNAs found to be differentially expressed by FDR. The GO analysis of the biological process of the target genes for miR-149-5p indicates that 164 (36.9%) of the genes are involved in cell signaling (*P* for enrichment = 5.28 × 10^−5^) and 166 (37.3%) in cell communication (*P* = 7.72 × 10^−5^; Table 3A). One hundred ninety-five (22.5%) of the target genes for miR-203a are involved in the negative regulation of cellular process (*P* for enrichment = 9.66 × 10^−5^), and many target genes are involved in the regulation of biosynthetic or cellular metabolic process (Table 3B). Ninety (20.2%) of the target genes for miR-222-3p are involved in nervous system development (*P* for enrichment = 1.30 × 10^−8^), and about one-third in cellular metabolic or biosynthetic process (Table 3C). The GO analysis of the biological process of the target genes for miR-122-5p failed to find significant enrichment of target genes involved in any biological process. The corresponding molecular function analysis indicates that 2 genes are involved in glucosamine-6-phosphate deaminase activity (*P* = 2.18 × 10^−2^; Table 3D).

**Table 3:**
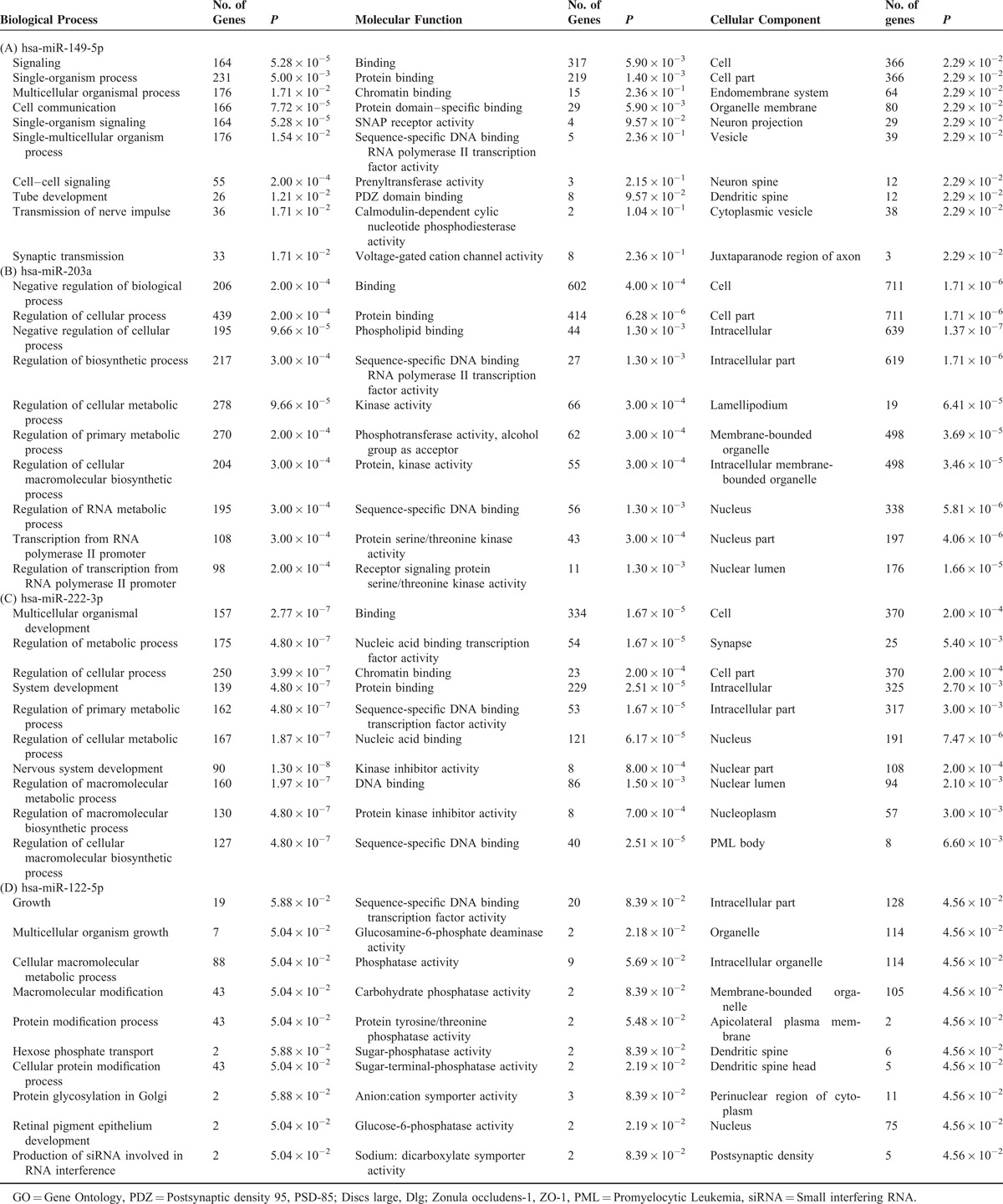
Top 10 Enriched GO Categories for Targeted Genes of hsa-miR-149-5p, hsa-miR-203a, hsa-miR-222-3p, and hsa-miR-122-5p

The KEGG pathway analysis of the target genes for miR-149-5p indicates that 14 genes (3.1%) are involved in mitogen-activated protein kinase (MAPK) signaling pathway (*P* = 6.74 × 10^−5^), and 9 (2.0%) in lysosome (*P* = 2.00 × 10^−4^; Table 4A). Twenty-three (2.6%) target genes for miR-203a are involved in insulin signaling pathway (*P* = 6.72 × 10^−13^), 25 (2.9%) in focal adhesion (*P* = 2.01 × 10^−11^), and 31 (3.6%) in cancer-related pathways (*P* = 3.51 × 10^−11^; Table 4B). Seventeen target genes (3.8%) for hsa-miR-222-3p are involved in cancer-related pathways (*P* = 4.44 × 10^−6^), 9 (2.0%) in ErbB signaling (*P* = 1.17 × 10^−5^), 11 (2.5%) in Wnt signaling (*P* = 1.26 × 10^−5^), and 13 (2.9%) in MAPK signaling (*P* = 9.29 × 10^−5^; Table 4C). Twelve target genes (7.0%) for miR-122-5p are involved in metabolic pathway (*P* = 0.012), 3 (1.7%) in antigen processing (*P* = 0.015), 2 (1.2%) in citrate cycle (*P* = 0.021), and 4 (2.3%) in endocytosis (*P* = 0.025; Table 4D).

**Table 4:**
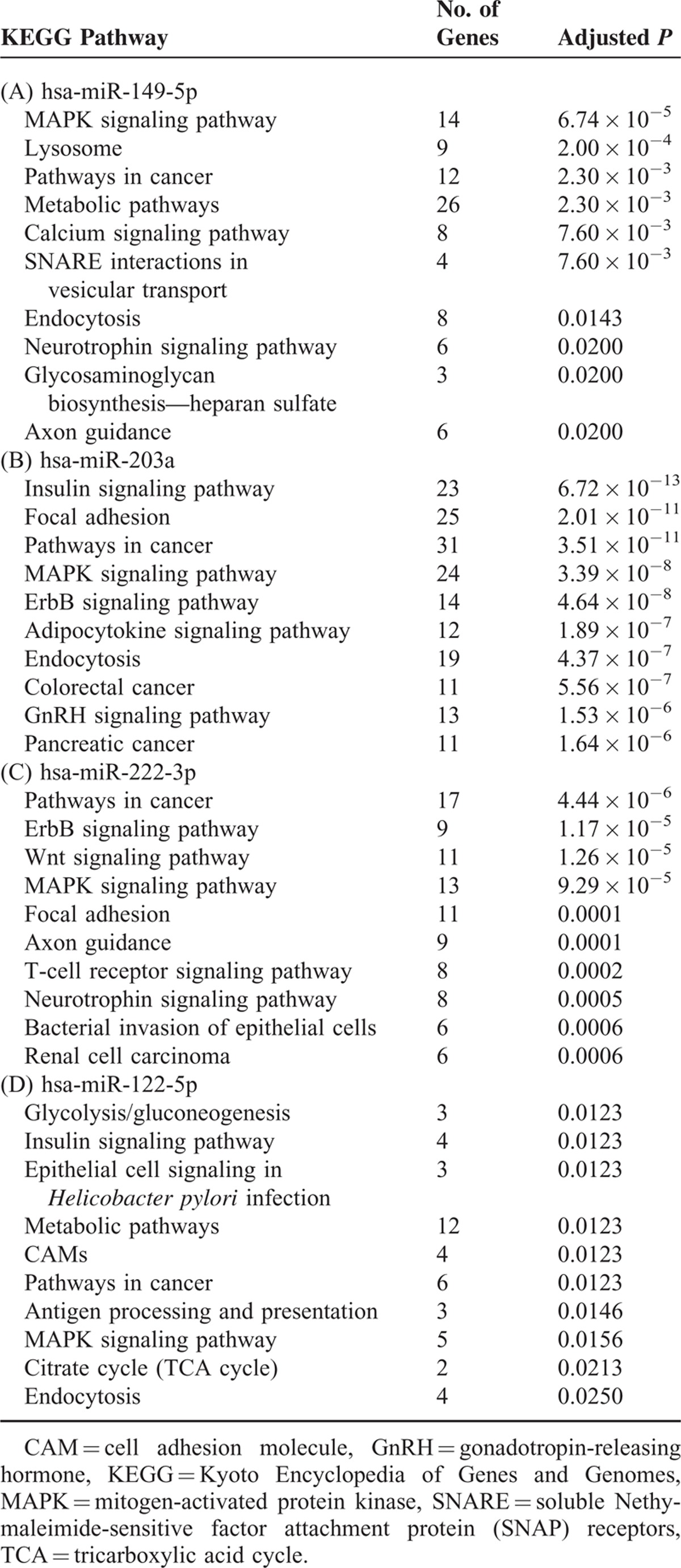
Top 10 Enriched KEGG Pathways for Targeted Genes of hsa-miR-149-5p, hsa-miR-203a, hsa-miR-222-3p, and hsa-miR-122-5p

**Figure 1:**
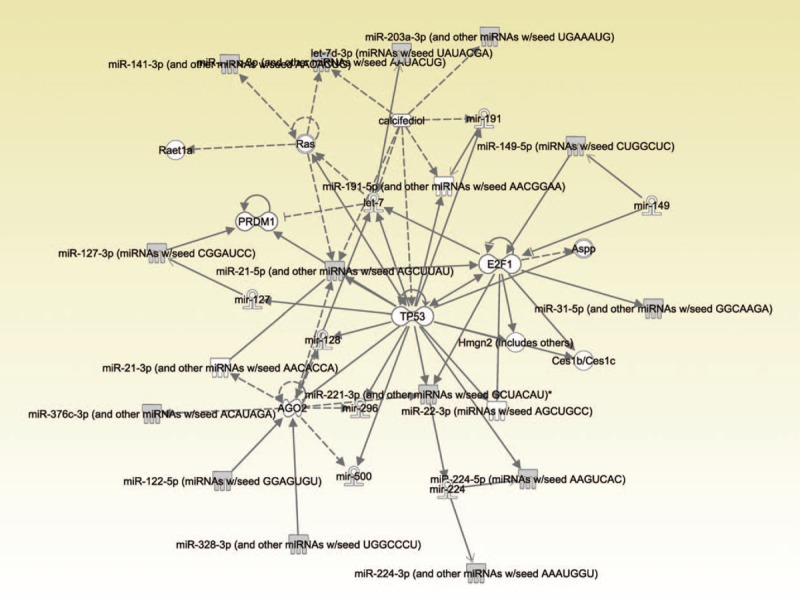
The network constructed by IPA based on miRNAs exhibiting significant changes in patients with scar as identified by DC-SIS. Solid and dotted lines indicate direct and indirect relationships, respectively. For more details about network shapes and types of relationships, please see http://www.biolreprod.org/content/suppl/2011/02/23/biolreprod.110.090019.DC1/90019SupLegend.pdf. DC-SIS = a sure independence screening procedure, IPA = Ingenuity Pathway Analysis, miRNA = microRNA.

IPA constructed 4 networks, and 1 network distinguished itself with a p-score of 39, compared with a score of 3 for the 3 other networks constructed by IPA. This network comprises 35 miRNAs/molecules including 14 miRNAs identified by DC-SIS (Figure 1). It centers on miR-21-5p, let-7, AGO2, tumor protein p53 (TP53), and E2F1. IPA also provided high-level biological functions associated with this network, which indicated that the identified network plays a critical role in cell death and survival (*P* = 1.06 × 10^−6^, data not shown).

## DISCUSSION

In this article, we conducted a pilot study to compare the miRNA expression of the scar and normal skin cells obtained from 9 patients suffering acute skin injuries. We identified 4 miRNAs showing significant change in miRNA expression. The target genes of the 4 miRNAs exhibit a variety of biological functions, including cell signaling and communication, biosynthetic or cellular metabolism, and nervous system development, and are involved in multiple pathways such as MAPK, Wnt signaling, and focal adhesion. We identified 1 network, comprising 14 focus miRNAs, which plays an important role in cell death and survival.

Wound healing is a complex process that involves hemostasis, inflammation, proliferation, and maturation.^9^ Previous research has identified multiple miRNAs exhibiting significant change in expression in the presence of acute injuries and explored their function in wound healing. miR-31 is highly expressed during wound healing, and transgenic mouse model with elevated miR31 levels showed aberrant wound healing and hair loss.^10^ In our study, we found that miR-31 is among the top-hit miRNAs (Table 2), and is upregulated in scar cells. miR-21-5p is also among top-hit miRNAs and upregulated, consistent with a previous study showing that miR-21 promotes keratinocyte migration and reepithelialization during wound healing in mice through tissue inhibitor of metalloproteinases 3 and T-cell lymphoma invasion and metastasis 1.^11^ In our study, miR-21-5p is also an important hub in the network we constructed, and this further implies that it might have important functions in wound healing.

Wound healing involves multiple steps and a variety of cells and events. Cell differentiation and proliferation is essential for tissue remodeling and recovery of physiologic functions.^12^ We identified multiple miRNAs involved in cell differentiation. One miRNA is miR-203, which has potent antiproliferative function and regulates the balance between stem cell proliferation and terminal differentiation in skin cells, possibly by targeting p63 when stem cells in the epidermis are proliferating and differentiating into stratified epithelium.^13^ miR-203 can be considered as a “skin-specific miRNA" as it has the highest level of expression in the skin.^14^ It induces cell cycle exit and represses “stemness" in epidermal progenitors, suggesting its involvement in keratinocyte differentiation.^13,15^ The other miRNA, miR-222, can stimulate cell proliferation via coordinating competency for initiation of S phase with growth factor signaling pathways.^16^ When elevated, it can prevent quiescence and induce precocious S-phase entry, and hence trigger cell death^16^ (we observed downregulation of miR-222 in patients suffering acute skin injuries). These results highlight the critical roles of miRNAs in regulating wound healing of the skin after acute injuries, and also call for future studies to carefully examine the exact mechanism through which they function in scar formation and skin remodeling.

We found that MAPK is a pathway in which the target genes of multiple top-hit miRNAs (miR-149 and miR-222; Table 4A and C) are involved. MAPKs participate in directing cellular responses to various stimuli such as heat shock,^17^ and regulate gene expression,^18^ cell differentiation, proliferation,^19^ and survival.^20^ Application of MAPK inhibitor promotes wound healing by stimulating early generation of connective tissues in the wounding area.^21^ Whether and how the identified miRNAs influence wound healing in the skin by regulating MAPK pathways warrants further investigation.

The TP53 is a tumor suppressor gene, and is essential in the regulation of cell proliferation.^22^ Previous research found that transient inhibition of TP53 can accelerate wound healing by promoting leukocyte recruitment, increasing cell proliferation, and reducing apoptotic cell death.^23^ miR-21 targets multiple components of TP53 (Figure 1), and its downregulation can lead to repression of growth, increased apoptosis, and cell cycle arrest.^24^ Consistent with these findings, we found that miR-21 exhibited upregulation in patients with scar (Table 2), implying that miR-21 might be implicated in cell proliferation and skin regeneration in patients with scar following serious injuries in the skin.

Wnt signals are essential for the development and homeostasis of the skin, and Wnt signaling is involved in multiple processes of wound healing following acute injuries.^25^ It is implicated in the reconstitution of the dermal compartment and in the construction of epithelial structures.^26^ Augmenting endogenous Wnt signaling could result in improved skin wound healing, suggesting the potential of applying liposomal Wnt3a for the therapeutic treatment of cutaneous wounds; however, the underlying mechanism remains poorly understood.^27^ miR-222-3p targets multiple genes involved in Wnt signaling, such as *DDK2* (dickkopf WNT signaling pathway inhibitor 2), *AXIN2* (axin 2), and *FRAT2* (frequently rearranged in advanced T-cell lymphomas 2). How miR-222-3p regulates its target genes involved in Wnt signaling, thereby influencing wound healing and scar formation in the skin, deserves further investigation.

Since 1 miRNA can target many genes sharing the same seeding sequence simultaneously, miRNAs have their advantages as therapeutic targets. Currently, miRNA manipulation can be achieved by either increasing the expression of a particular miRNA with synthetic miRNA mimics or blocking its action with chemically engineered oligonucleotides called antagomirs.^28^ The identified miRNAs, if confirmed by future studies, may shed light on the development of efficient therapies for wound healing of the patients suffering acute skin injuries.

Our study has some limitations. With limited sample size, we have limited power in detecting miRNAs showing significant changes in expression. However, we believe that the miRNAs identified by all of the 3 statistical methods represent potential markers in wound healing and deserve further investigation. Our study is cross-sectional, and, therefore, we cannot analyze trajectories of miRNA expression during the wound healing process after acute skin injuries. In this study, we focused on the hypertrophic scar, which is different from keloids in that a hypertrophic scar can occur anywhere where there is a traumatic injury or operative incision.^29^ Although skin tissue varies greatly with age, gender, and location of body, in this study normal skin specimen was taken from around the scar area of the same patient to minimize confounding factors.

In summary, in this pilot study, we screened for miRNAs exhibiting differential expression in patients suffering acute injuries in the skin. We identified 4 miRNAs showing significant changes in expression. Their target genes are involved in a variety of biological functions, particularly cell signaling and communication, biosynthetic, or cellular metabolism. These target genes are involved in multiple pathways such as MAPK, Wnt signaling, and focal adhesion. We also constructed a network showing the biological and chemical interaction of the focus miRNAs. The exact functions of the identified miRNAs remain unknown and deserve further investigation. Future studies with larger sample size are also needed to validate the findings of this pilot study.

## References

[1] Shilo S Roy S Khanna S MicroRNA in cutaneous wound healing: a new paradigm. *DNA Cell Biol* 2007; 26:227–237. 17465889 10.1089/dna.2006.0568

[2] Eming SA Krieg T Davidson JM Inflammation in wound repair: molecular and cellular mechanisms. *J Invest Dermatol* 2007; 127:514–525. 17299434 10.1038/sj.jid.5700701

[3] Calin GA Sevignani C Dumitru CD Human microRNA genes are frequently located at fragile sites and genomic regions involved in cancers. *Proc Natl Acad Sci U S A* 2004; 101:2999–3004. 14973191 10.1073/pnas.0307323101 PMC365734

[4] Mavrakis KJ Van Der Meulen J Wolfe AL A cooperative microRNA-tumor suppressor gene network in acute T-cell lymphoblastic leukemia (T-ALL). *Nat Genet* 2011; 43:673–678. 21642990 10.1038/ng.858 PMC4121855

[5] Ooi CH Oh HK Wang HZ A densely interconnected genome-wide network of microRNAs and oncogenic pathways revealed using gene expression signatures. *PLoS Genet* 2011; 7:e1002415. 22194702 10.1371/journal.pgen.1002415 PMC3240594

[6] Mills SJ Cowin AJ MicroRNAs and their roles in wound repair and regeneration. *Wound Pract Res* 2013; 21:26–40.

[7] Bolstad BM Irizarry RA Astrand M A comparison of normalization methods for high density oligonucleotide array data based on variance and bias. *Bioinformatics* 2003; 19:185–193. 12538238 10.1093/bioinformatics/19.2.185

[8] Li R Zhong W Zhu L Feature screening via distance correlation learning. *J Am Stat Assoc* 2012; 107:1129–1139. 25249709 10.1080/01621459.2012.695654 PMC4170057

[9] Wang T Feng Y Sun H miR-21 regulates skin wound healing by targeting multiple aspects of the healing process. *Am J Pathol* 2012; 181:1911–1920. 23159215 10.1016/j.ajpath.2012.08.022

[10] Botchkareva NV MicroRNA/mRNA regulatory networks in the control of skin development and regeneration. *Cell Cycle* 2012; 11:468–474. 22262186 10.4161/cc.11.3.19058

[11] Yang X Wang J Guo SL miR-21 promotes keratinocyte migration and re-epithelialization during wound healing. *Int J Biol Sci* 2011; 7:685–690. 21647251 10.7150/ijbs.7.685 PMC3107477

[12] Braiman-Wiksman L Solomonik I Spira R Novel insights into wound healing sequence of events. *Toxicol Pathol* 2007; 35:767–779. 17943650 10.1080/01926230701584189

[13] Yi R Poy MN Stoffel M A skin microRNA promotes differentiation by repressing ‘stemness’. *Nature* 2008; 452:225–229. 18311128 10.1038/nature06642 PMC4346711

[14] Sonkoly E Wei T Janson PC MicroRNAs: novel regulators involved in the pathogenesis of psoriasis? *PLoS One* 2007; 2:e610. 17622355 10.1371/journal.pone.0000610 PMC1905940

[15] Lena AM Shalom-Feuerstein R Rivetti di Val Cervo P miR-203 represses ‘stemness’ by repressing DeltaNp63. *Cell Death Differ* 2008; 15:1187–1195. 18483491 10.1038/cdd.2008.69

[16] Medina R Zaidi SK Liu CG MicroRNAs 221 and 222 bypass quiescence and compromise cell survival. *Cancer Res* 2008; 68:2773–2780. 18413744 10.1158/0008-5472.CAN-07-6754 PMC3613850

[17] Gorostizaga A Brion L Maloberti P Heat shock triggers MAPK activation and MKP-1 induction in Leydig testicular cells. *Biochem Biophys Res Commun* 2005; 327:23–28. 15629424 10.1016/j.bbrc.2004.11.129

[18] Sassone-Corsi P Mizzen CA Cheung P Requirement of Rsk-2 for epidermal growth factor-activated phosphorylation of histone H3. *Science* 1999; 285:886–891. 10436156 10.1126/science.285.5429.886

[19] Cowley S Paterson H Kemp P Activation of MAP kinase kinase is necessary and sufficient for PC12 differentiation and for transformation of NIH 3T3 cells. *Cell* 1994; 77:841–852. 7911739 10.1016/0092-8674(94)90133-3

[20] Bonni A Brunet A West AE Cell survival promoted by the Ras-MAPK signaling pathway by transcription-dependent and -independent mechanisms. *Science* 1999; 286:1358–1362. 10558990 10.1126/science.286.5443.1358

[21] Shurygina IA Shurygin MG Granina GB Application of mitogen-activated protein kinase inhibitor SP 600125 for wound healing control. *J Regen Med Tissue Eng* 2012; 6:232–237.

[22] Mendoza-Rodriguez CA Cerbon MA Tumor suppressor gene p53: mechanisms of action in cell proliferation and death. *Rev Invest Clin* 2001; 53:266–273. 11496714

[23] Vollmar B El-Gibaly AM Scheuer C Acceleration of cutaneous wound healing by transient p53 inhibition. *Lab Invest* 2002; 82:1063–1071. 12177245 10.1097/01.lab.0000024363.37866.45

[24] Papagiannakopoulos T Shapiro A Kosik KS MicroRNA-21 targets a network of key tumor-suppressive pathways in glioblastoma cells. *Cancer Res* 2008; 68:8164–8172. 18829576 10.1158/0008-5472.CAN-08-1305

[25] Whyte JL Smith AA Helms JA Wnt signaling and injury repair. *Cold Spring Harb Perspect Biol* 2012; 4:a008078. 22723493 10.1101/cshperspect.a008078 PMC3405869

[26] Bielefeld KA Amini-Nik S Alman BA Cutaneous wound healing: recruiting developmental pathways for regeneration. *Cell Mol Life Sci* 2013; 70:2059–2081. 23052205 10.1007/s00018-012-1152-9 PMC3663196

[27] Whyte JL Smith AA Liu B Augmenting endogenous Wnt signaling improves skin wound healing. *PLoS One* 2013; 8:e76883. 24204695 10.1371/journal.pone.0076883 PMC3799989

[28] Schneider MR MicroRNAs as novel players in skin development, homeostasis and disease. *Br J Dermatol* 2012; 166:22–28. 21824129 10.1111/j.1365-2133.2011.10568.x

[29] Kischer CW Shetlar MR Chvapil M Hypertrophic scars and keloids: a review and new concept concerning their origin. *Scan Electron Microsc* 1982; 1699–1713. 7184146

